# Diagnosis and Management of Attention-Deficit/Hyperactivity Disorder: A Practitioner’s Perspective

**DOI:** 10.3390/jcm14092874

**Published:** 2025-04-22

**Authors:** Mansour M. Alotaibi, Naif Z. Alrashdi, Sultan A. Alanazi, Marzouq K. Almutairi, Bakriah Y. Alzubaidi, Maraheb M. Alkhalidi, Deemah Alateeq, Mohammed M. Alqahtani

**Affiliations:** 1 Department of Rehabilitation, Faculty of Applied Medical Sciences, Northern Border University, Arar 73213, Saudi Arabia; 2 King Salman Center for Disability Research, Riyadh 11614, Saudi Arabia; 3 Department of Physical Therapy and Health Rehabilitation, College of Applied Medical Sciences, Majmaah University, Al-Majmaah 11952, Saudi Arabia; n.alrashedy@mu.edu.sa (N.Z.A.); sa.alanazi@mu.edu.sa (S.A.A.); 4 Health and Basic Sciences Research Center, Majmaah University, Al Majma’ah 11952, Saudi Arabia; 5 Department of Physical Therapy, College of Applied Medical Sciences, Qassim University, Buraydah 51452, Saudi Arabia; mk.almutairi@qu.edu.sa; 6 Department of Rehabilitation Medicine, King Salman Specialist Hospital, Ministry of National Guard Health Affairs, Taif 11426, Saudi Arabia; pakriahya@gmail.com; 7 Department of Community Medicine and Behavioral Science, Faculty of Medicine, Kuwait University, Jabriya 13009, Kuwait; maraheb.alkhalidii@gmail.com; 8 Internal Medicine Department, College of Medicine, Princess Nourah Bint Abdulrahman University, P.O. Box 84428, Riyadh 11671, Saudi Arabia; daalateeq@pnu.edu.sa; 9 Department of Respiratory Therapy, College of Applied Medical Sciences, King Saud Bin Abdulaziz University for Health Sciences, Riyadh 11481, Saudi Arabia; qahtanimoh@ksau-hs.edu.sa; 10 King Abdullah International Medical Research Center, Riyadh 11481, Saudi Arabia

**Keywords:** ADHD, barriers, assessment, stimulants

## Abstract

**Background/Objective:** Attention-deficit/hyperactivity disorder (ADHD) is a common neurodevelopmental disorder in Saudi Arabia, yet challenges remain in diagnosis and treatment accessibility. This study examined healthcare practitioners’ clinical approaches to ADHD diagnosis and management in Saudi Arabia and identified gaps in practice. **Methods:** This cross-sectional study included healthcare practitioners working in various healthcare settings across Saudi Arabia. The sample included psychiatrists, pediatricians, psychologists, and other relevant specialists. Clinical practices regarding ADHD diagnosis, the use of ADHD screening tools, adherence to the Diagnostic and Statistical Manual of Mental Disorders (DSM-5) criteria, treatment strategies, medication prescription protocols, and reported challenges were investigated. **Results:** A total of 43 licensed healthcare practitioners with a minimum of 2 years of clinical experience managing ADHD participated. Among participants, 81.4% were psychiatrists, and 53.5% managed ADHD cases in children/adolescents exclusively. Approximately 86.0% of the sample used screening tools, with the Vanderbilt Assessment Scale being the most used (67.6%). However, only 55.8% addressed all 18 DSM-5 ADHD symptoms during the evaluation. Combined pharmacological and non-pharmacological treatment approaches (74.4%) were preferred. Interestingly, only 7.0% prescribed amphetamine-based stimulants due to the lack of clear guidelines. Key barriers included a lack of early screening programs (65.1%), limited ADHD medication option availability (51.2%), and extended referral waiting lists (44.2%). **Conclusions:** Significant variability in ADHD diagnosis and treatment practices was evident among healthcare providers in Saudi Arabia. Specifically, a large proportion of healthcare providers do not fully comply with the standard DSM-5 diagnosis criteria. Major barriers to ADHD diagnosis and treatment in Saudi Arabia include extended referral waiting lists, limited availability of psychostimulant medications, and standardized clinical guidelines. A national ADHD protocol must be advocated, and access to diverse treatment options should be improved.

## 1. Introduction

Attention/deficit hyperactivity disorder (ADHD) is a common neurodevelopmental disorder that affects 5–8% of children and adolescents globally [1,2], with 2.6% of cases persisting into adulthood [3]. ADHD can be categorized into three subtypes: predominantly inattentive, predominantly hyperactive/impulsive, or combined [4]. The global prevalence of adult-diagnosed ADHD is estimated at 6.8%, reflecting a growing recognition of this diagnosis in adults [3]. In Saudi Arabia, a national survey of mental disorders estimated the prevalence of ADHD in adolescents and adults at 11.3% [5]. Another systematic review and meta-analysis suggested that ADHD prevalence was 12.4% [6]. However, ADHD prevalence among children in Saudi Arabia remains unclear.

Major ADHD symptoms, such as inattention and/or hyperactivity-impulsivity, could lead to significant changes in personal life, leading to academic underachievement [7,8], unemployment, impaired professional performance [9], and/or poor quality of life [10,11,12]. A reliable and valid diagnosis of ADHD involves a comprehensive and structured process that typically assesses affected individuals along with their caregivers, parents, and teachers, including the extent and frequency of symptoms as well as concurrent conditions [13]. Different organizations have established clinician practice guidelines (CPGs) to assist practitioners in selecting the most appropriate method for evaluating and managing ADHD [14]. The most commonly accepted approach for ADHD diagnosis is a detailed clinical interview, as recommended by the American Academy of Pediatrics (AAP) [15] and the National Institute for Health and Clinical Excellence (NICE) [16].

Furthermore, a deeper examination of contextual and systemic factors influencing ADHD assessment can help bridge the gap between symptom recognition and accurate diagnosis. Questionnaires and psychological assessment tools may also assist with conducting professional evaluation, which can be used to aid ADHD diagnosis based on medical history and a minimum of one reference (e.g., parent, caregiver) [17]. In Saudi Arabia, a national study involving more than 4000 participants for mental disorders, including ADHD, used clinical interviews for the diagnosis [18], assuring compliance with international guidelines [15,16]. However, deviations from confirmed clinical diagnosis and reliance on self-reported questionnaires occur, potentially leading to inaccurate measurements and estimations of ADHD prevalence [19] and ADHD comorbidity [20]. Further, a systematic review of 334 studies reported that ADHD is overdiagnosed in children and adolescents [21], which is concerning in terms of educational performance and overuse of psychostimulants, particularly among those with milder symptoms [22]. To these ends, it is essential to evaluate current ADHD diagnostic practices in Saudi Arabia to better understand the qualifications necessary for diagnosis and the training required for the effective diagnosis and treatment of ADHD.

For ADHD treatment, most international CPGs recommend parent-training programs before offering medications that mainly include methylphenidate-derived psychostimulants for young children (ages 4–6) [15,16]. For older children (ages 6–12) and adolescents (ages 12–18), a combination of medication and training and/or behavioral intervention is strongly recommended [15,16]. For adults, offering medication if symptoms are causing significant problems is strongly recommended [15,16]. In addition to medications, cognitive-behavioral therapy and exercise have emerged as non-pharmacological treatments for reducing ADHD-related symptoms [23,24], thereby reducing the demand on pharmacological treatment [25]. Experts have developed the Saudi Ministry of Health (MOH) [26] protocol for ADHD diagnosis and treatment in Saudi Arabia derived from six CPGs, including the AAP [15] and NIC [16]. Further, researchers led a national initiative to adopt the NICE guidelines in a single center in 2019 [27], yet implementation of these standards is limited. Adherence to standard care requires further work to minimize variations in practice, which could lead to ADHD overdiagnosis and/or suboptimal treatment [21].

The absence of locally adopted, evidence-based clinical practice guidelines (CPGs) for ADHD treatment has resulted in the lack of clearance for several internationally recommended medications by the Saudi Food and Drug Authority (SFDA), specifically, amphetamine-derived psychostimulants, such as mixed amphetamine salts (Adderall) [28]. Data from pharmaceutical sales also highlight a slight increase in the consumption of methylphenidate-derived stimulants in Saudi Arabia [29]. Nevertheless, there is a critical need for a broader range of medication options available to physicians.

Despite the growing recognition of ADHD as a public health priority, there remains a critical gap in the literature regarding healthcare practitioners’ real-world experiences in diagnosing and managing ADHD in Saudi Arabia. Most local research has focused on prevalence and patient knowledge, while empirical investigations of practitioner behavior, decision-making, and access challenges remain sparse. Furthermore, the absence of a unified national protocol may contribute to wide variability in treatment approaches. This study addresses these gaps by examining the perspectives of Saudi-based healthcare professionals on diagnostic practices, treatment barriers, and medication availability. The hypothesis was that there would be variability in practice and a shortage of stimulant medication classes accessible to physicians.

## 2. Methods

We used a cross-sectional design to evaluate and understand how ADHD is diagnosed and managed in Saudi Arabia, focusing only on in-charge HCP practitioners’ perspectives who directly manage individuals with ADHD. This current study obtained its ethical approval from the Northern Border University Institutional Review Board (IRB Approval: HAP-09-A-043), and all participants provided written informed consent before participation in the study.

### 2.1. Participants and Study Design

Participants were included if they were licensed healthcare professionals (HCPs) with clinical expertise in treating ADHD, had a minimum of two years of post-qualification clinical experience in working with ADHD patients, and were proficient in English. HCPs may include the following clinical specialties: family medicine specialists, pediatricians, psychiatrists, developmental-behavioral pediatricians, psychologists, or other licensed physicians actively diagnosing and treating ADHD. Participants were excluded if they were studying, practicing, or receiving training outside of Saudi Arabia at the time of the study and/or had less than two hours of weekly clinical practice involving ADHD.

We attempted to recruit HCPs from various geographic locations for all professions and work settings in Saudi Arabia. Our recruitment process was completed using a purposeful and snowball sampling technique, and this non-probability sampling method yielded a convenient sample. Briefly, this method relies on existing participants to recruit additional subjects from their acquaintances, resembling a rolling snowball that gathers participants as it spreads across the population. Enrollment of the study participants began in June and ended in November 2024. Additionally, different recruitment strategies, such as social media (e.g., X platform), the authors’ professional networks, and relevant societies and organizations, were implemented to maximize our sample size. The recruited HCPs were asked to recommend any potential HCPs with clinical expertise in working with ADHD to participate in this study. 

### 2.2. ADHD Survey

An online questionnaire was created and used to reflect the HCPs’ current practices for screening, diagnosing, treating, and managing ADHD in Saudi Arabia. This study’s questionnaire comprised three main sections.

The first section focused on demographic data to describe our sample. This section included questions regarding age, sex, profession, the primary age group of patients that HCPs see in their clinics, years of experience in managing individuals with ADHD, work settings (e.g., primary care), level of care provided at their workplace, the region in Saudi Arabia where they currently practice managing ADHD, and whether HCPs had completed any specific training programs/courses related to ADHD conditions.

The second section’s questions aimed to explore the participants’ practice and diagnostic procedures for individuals with ADHD. This section included data regarding tools used to screen for ADHD symptoms, specific questions asked during ADHD screening, whether HCPs conduct clinical interviews for evaluation with their patients, parents, or caregivers, and whether HCPs address all 18 ADHD symptoms listed in the DSM-5 during clinical interviews. Furthermore, this section pertains to questions about the topics HCPs discuss regarding ADHD symptoms with patients or their parents during clinical interviews and the average number of ADHD cases they screen each week.

The third section focused on understanding treatment strategies and the challenges that HCPs encounter while managing individuals with ADHD in Saudi Arabia. All participants were asked about the medications they prescribe for managing ADHD symptoms, other treatment options offered to them, and the frequency of follow-up appointments they are required to have. Additionally, HCPs were asked if they refer their ADHD patients to rehabilitation services. For those who often refer their patients to rehabilitation teams, we asked them about the primary reasons for the referrals, such as addressing physical injuries or musculoskeletal pain.

### 2.3. Data Analysis

The data were analyzed using IBM^®^ SPSS Statistics for Windows, Version 27.0 (IBM Corp., Armonk, NY, USA). The statistical significance level was accepted as α = 0.05 for all statistical tests. All the variables were evaluated using the Shapiro–Wilk and Kolmogorov–Smirnov tests for normality distribution. This indicated no violation of the normality assumption of each variable entered into the analyses. Descriptive analysis was utilized to calculate the frequencies and proportions of the participant characteristics as categorical variables (i.e., sex, professional backgrounds, years of experience, primary practice, place of current practice, level of care, and the sector of current practice) and was expressed as count (n) and percentage (%) and as mean and standard deviation (mean ± SD) for continuous variables (i.e., age). The Chi-square (ꭓ^2^) test was used to assess whether the gender distribution differed across categories of participant characteristic variables. Furthermore, the independent *t*-test was used to compare the means of age and determine if there was a significant difference between the two genders. In addition to that, descriptive analysis was chosen to summarize the survey questions related to screening for ADHD and their treatment as count (n) and percentage (%).

## 3. Results

### 3.1. Demographic and Clinical Characteristics

The total number of enrolled HCPs in the current study was 43, which included 25 men (58.1%) and 18 women (41.9%) with a mean age of 34.8 ± 9.0 years (Table 1). Approximately 81.4% of participants were psychiatrists, with men significantly more likely to work in the psychiatric field compared to women (96.0%; *p* = 0.010). Participants reported that their primary practice area included either children, adolescents, or both (n = 23; 53.5%) or a combination of children, adolescents, and adults (n = 20; 46.5%) with ADHD. Nearly half of the participants had up to two years of clinical experience managing patients with ADHD (44.2%). Among the study participants, 53.5% were from the Central region, whereas 25.6% were from the Western region of Saudi Arabia. Male HCPs had a higher rate of employment in the governmental sector than female HCPs (96.0%; *p* = 0.026). Although the *p*-values were statistically significant for profession and sector, the sample size was relatively small (n = 43), which may exaggerate effect sizes. The gender skew in professional roles and sector distribution may reflect broader trends in workforce representation or sociocultural preferences in the region, potentially influencing how ADHD is managed across genders and settings.

### 3.2. Survey Questions Response

The results of this descriptive analysis of the responses to the survey questions regarding ADHD screening are shown in Table 2. Over half of the participants (53.5%) reported receiving specific training for the diagnosis and/or treatment of ADHD. The majority of participants considered using a tool to screen for ADHD symptoms (86.0%). Regarding ADHD screening tools, 67.6%, 54.1%, and 16.2% of HCPs were using the Vanderbilt Assessment Scale, Conners Rating Scales, and Child Behavior Checklist, respectively. Most participants considered asking about major ADHD symptom domains (e.g., inattention and hyperactivity/impulsivity) during their screening interview (88.4%) and ensured whether these symptoms were causing impairment (81.4%). Approximately 55.8% of HCPs evaluated their ADHD cases based on the 18 symptoms listed in the DSM-5 with patients or their parents. During the clinical interviews, 65.1% of HCPs discussed all ADHD symptoms with the parent or patient, including the number of symptoms, their duration, age at onset, and the setting (e.g., home, school). Furthermore, 60.5% of HCPs screened on average one to five patients for ADHD each week (Table 2).

Most clinicians followed best practices by utilizing proper screening tools and involving caregivers. However, since only about half assessed all 18 DSM-5 symptoms, some children may not receive a comprehensive evaluation, risking incomplete or inaccurate diagnoses. The noticeable gap between clinicians who fully assessed all DSM-5 ADHD symptoms and those who did not (55.8% vs. 44.2%) indicates a moderate practical effect, with significant implications for diagnostic accuracy and the effectiveness of treatment plans.

In response to questions regarding the treatment of ADHD cases, HCPs reported the most common challenges included the lack or absence of early screening ADHD programs (65.1%), the absence of available medication on the physician desk (51.2%), family-related factors (46.5%), and long referral waiting lists (44.2%). Moreover, 55.8% of HCPs always requested follow-up appointments for ADHD cases (Table 3). Additionally, 39.5% of HCPs rarely referred ADHD cases to rehabilitation services (e.g., physical therapy or occupational therapy), whereas 18.6% of HCPs did so frequently. One of the main reasons for referring ADHD cases to rehabilitation services was for the management of ADHD symptoms only (51.2%). For the class of medication, stimulants (methylphenidate and amphetamines) were reported to be the most often prescribed medication by HCPs for ADHD (81.4%; Table 3). Amphetamine-derived stimulants were prescribed by 7.0% of the sample. The discrepancy between stimulant use (81.4%) and amphetamine use (7%) suggests considerable practical and clinical distinctions in medication preferences, likely due to regulatory or cultural prescribing norms. The most commonly used treatment approach utilized by HCPs for ADHD patients was a combination of pharmacological and non-pharmacological treatment (74.4%), followed by pharmacological treatment only (20.9%; Table 3).

## 4. Discussion

According to our study findings, there were variations in the clinical practices related to ADHD diagnosis and treatment in Saudi Arabia, though most of our sample reported adherence to the international ADHD standards reported in CPGs and the Saudi MOH protocol [15,16,30], specifically concerning the use of clinical interviews and diagnostic tools. However, variations in the application of structured procedures existed and highlighted significant gaps in standardizing ADHD management. Our findings are consistent with the current literature, which highlights that disparities in ADHD diagnosis and treatment are influenced by resource availability and guideline adherence [31], potentially contributing to overdiagnosis and overtreatment [21]. Our findings call for the need for localized efforts to bridge gaps and improve consistency in clinical practice related to ADHD diagnosis and management in Saudi Arabia.

Only 55.8% of our participants conducted ADHD evaluations according to the DSM-5 criteria, potentially leading to inaccuracy in ADHD diagnosis [4]. DSM-5 evaluations require detailed symptom assessments in various settings, which can often be tedious in a busy clinic due to time constraints. Thus, many practitioners prefer quick screening scales, like the Vanderbilt Assessment Scale, over full DSM-5 assessments. Furthermore, the underutilization of comprehensive screening tools may be linked to limited practitioner training and resource constraints. This was evident by the fact that more than 44.2% of practitioners did not ask about all 18 DSM-5 symptoms for ADHD as part of their evaluation. In addition, clinical challenges such as the lack of early ADHD screening programs and limited access to medications were frequently reported by our sample in line with previous literature [18,21], highlighting systemic barriers in ADHD management. Our findings emphasize the importance of a more coordinated national strategy to enhance compliance with standard care for ADHD.

Screening practices for ADHD symptoms varied among our participants in this study, with the Vanderbilt Assessment Scale [32] being the most utilized, as recommended by the Saudi MOH protocol [26], followed by the Conners Rating Scales [33]. Nevertheless, approximately 15% of our sample did not use any screening tools for ADHD symptoms, raising significant concerns about inconsistencies in clinical practice. This finding is not surprising and aligns with global concerns regarding overdiagnosis and misdiagnosis when standardized protocols are not uniformly followed for ADHD [21,34]. In Europe, for example, variations in ADHD screening and diagnosis practices have been reported, leading to potentially inflated ADHD diagnosis rates in some countries such as Italy and the U.K. [35]. In Saudi Arabia, the lifetime prevalence of ADHD is estimated at 11.3% to 12.4% [5,6], which is higher than the global ADHD estimates (5–8%) [1,2]. Collectively, these challenges highlight the need for standardizing screening and diagnosis practices to minimize the likelihood of ADHD overdiagnosis.

The lack of available screening tools in Arabic could have contributed to the variation in practice found in this current study. Researchers have conducted forward and backward translation of the Vanderbilt Diagnostic Rating Scale into Arabic [36] and validation of two versions of the Arabic translation of the Vanderbilt ADHD Diagnostic Scale: the Vanderbilt ADHD Diagnostic Parent Rating Scale and the Vanderbilt ADHD Diagnostic Teacher Rating Scale [37]. Other researchers have translated Conner’s Parent–Teacher Rating Scale into Arabic and conducted reliability and validity evaluations [38]. However, these tools lack cultural adaptation for the populations of Saudi Arabia, which limits their usability for clinicians. Therefore, more efforts are needed to translate, culturally adapt, and validate standard ADHD tools for the ADHD population in Saudi Arabia.

Clinical interviews with ADHD patients and their parents, custodians, or caregivers, if needed, are essential for diagnosis and management [39,40]. Our findings documented a high compliance with this practice, with more than 75% of the sample. Family involvement in healthcare decisions in Saudi Arabia is highly valued, which may encourage greater parental participation in clinical assessments. In addition, more than 30% of the sample reported missing asking about the number of symptoms, duration, age at onset, or setting (e.g., home, school) with the patient or parent. Furthermore, more than 44% of them did not often ask about all ADHD symptoms listed in the DSM-5. Therefore, further training on proper clinical interviewing with patients and their parents in accordance with the DSM-5 guide is essential to enhance the standardization of ADHD evaluation.

Participants in this study reported significant barriers to ADHD treatment, particularly the limited availability of medications on the physician’s desk and lengthy referral waiting lists. This issue was accompanied by the lack of care provision for patients with ADHD in the primary care setting. Additionally, the lack of amphetamine-based psychostimulants in Saudi Arabia further restricted treatment options, as these medications are commonly used in ADHD treatment [41]. Only three participants in our study reported prescribing amphetamine-based psychostimulants for managing ADHD symptoms, even though the Saudi Food and Drug Administration did not authorize using these medications for ADHD. This practice emphasizes the need for further approvals for such medications due to the critical need to manage ADHD symptoms for this population. International CPGs, including AAP [15] and NICE [16], recommend initiating pharmacological treatment for children over the age of six and adolescents when ADHD symptoms cause significant functional impairment. For adults, psychostimulant medication is generally indicated when symptoms substantially interfere with social, academic, or occupational functioning and when non-pharmacological interventions alone are insufficient. Methylphenidate is typically first-line due to its safety profile, but amphetamine-based stimulants may be considered when symptom control is suboptimal. Thus, efforts should be made to advocate for the clearance of a broader range of amphetamine-based psychostimulants for ADHD treatment in Saudi Arabia.

Diversification of ADHD medication improves treatment adherence and symptom management, further emphasizing the need for policy changes. Addressing these systemic barriers/regulations requires coordinated efforts between policymakers, healthcare providers, and the pharmaceutical industry to ensure adequate access to a broad range of treatment options. Moreover, increasing public awareness and parental education could mitigate family-related barriers, improving the overall effectiveness of ADHD interventions.

Referrals of ADHD cases to rehabilitation services such as occupational and physical therapy were infrequent, with only one-third of our participants referring their ADHD patients. This practice indicates a missed opportunity for holistic ADHD management, particularly for addressing sensory processing issues and coexisting conditions commonly associated with ADHD [42]. Incorporating multidisciplinary approaches could enhance outcomes for individuals with ADHD. International guidelines emphasize the importance of multidisciplinary care in improving educational and behavioral outcomes for children with ADHD. Furthermore, fostering collaborations between healthcare providers, educators, and rehabilitation professionals could streamline referral processes and ensure timely access to supportive therapies. Establishing integrated care models and enhancing communication across disciplines could further optimize ADHD management.

In terms of practical and real-world significance, the data show a high reliance on pharmacological treatment for ADHD, with most clinicians prescribing stimulant medications. Encouragingly, many also incorporate non-pharmacological interventions, reflecting an integrated approach to care. However, the low rates of referral to rehabilitation services such as occupational or physical therapy may suggest a lack of awareness among providers or limited access to these services. This represents a missed opportunity to deliver more comprehensive, multidisciplinary care. Additionally, systemic barriers such as extended referral waiting lists and inadequate resources significantly hinder timely and effective service delivery, ultimately affecting patient outcomes.

The findings from this current study emphasize the urgent need to advocate for an MOH protocol tailored to ADHD care in Saudi Arabia. Integrating training programs can address inconsistencies in diagnosis and treatment. Additionally, enhancing medication availability and developing early screening programs could mitigate systemic challenges. Collaborative efforts involving governmental and private sectors are pivotal in achieving these objectives. Lessons from different countries with well-established ADHD programs, such as the U.S. and the U.K., can guide the development and implementation of Saudi-specific ADHD strategies. Moreover, leveraging technology, such as telemedicine and digital health tools, could support widespread training and access to ADHD care, particularly in remote or underserved areas.

Several limitations should be acknowledged in the current study. First, the sample size may not fully represent the entire clinical practice across Saudi Arabia. Second, the reliance on self-reported data introduces the possibility of reporting bias, which could not be avoided due to the study design. Third, using a cross-sectional study design prevents causal inferences about the observed associations. Future studies with larger, more representative samples and longitudinal designs are necessary to validate our findings.

## 5. Conclusions

This study provided a comprehensive overview of the current practices and challenges in ADHD care in Saudi Arabia. The findings underscored significant gaps in standardizing ADHD diagnosis and treatment, suggesting the need for national training in existing protocols and systemic improvements. These findings are valuable for healthcare policymakers, clinicians, and decision-makers aiming to improve ADHD diagnosis and treatment by addressing inconsistencies in clinical practice, medication accessibility, and diagnostic processes. Addressing these gaps through collaborative efforts and policy changes can enhance care delivery and outcomes for individuals with ADHD. Policymakers and healthcare providers should prioritize multidisciplinary collaboration and training to ensure a holistic approach to ADHD management.

## Figures and Tables

**Table 1:** Summary of participants’ demographic and clinical characteristics.

		Gender Distribution		
	Total Sample % (n)	Male % (n)	Female % (n)	*p*-Value *
Total	100.0 (43)	58.1 (25)	41.9 (18)	-
Age (mean ± SD)	34.8 ± 9.0	33.7 ± 8.5	36.3 ± 9.8	0.365
Profession				
Psychiatrist	81.4 (35)	96.0 (24)	61.1 (11)	**0.010**
Psychologist	7.0 (3)	4.0 (1)	11.1 (2)	
Others (e.g., Pediatrician, Counselor)	11.6 (5)	0.0 (0)	27.8 (5)	
Primary practice				
Children/Adolescents	53.5 (23)	60.0 (15)	44.4 (8)	0.313
Children/Adolescents/Adults	46.5 (20)	40.0 (10)	55.6 (10)	
Years of experience with ADHD practice				
>2 years	44.2 (19)	48.0 (12)	38.9 (7)	0.199
2–5 years	25.6 (11)	32.0 (8)	16.7 (3)	
>5 years	30.2 (13)	20.0 (5)	44.4 (8)	
Place of current practice (Regions)				
Central	53.5 (23)	64.0 (16)	38.9 (7)	0.265
Western	25.6 (11)	20.0 (5)	33.3 (6)	
Eastern/Southern	20.9 (9)	16.0 (4)	27.8 (5)	
Level of care				
Tertiary healthcare	69.8 (30)	76.0 (19)	61.1 (11)	0.339
Secondary healthcare/Private practice	20.9 (9)	20.0 (5)	22.2 (4)	
Primary health care	9.3 (4)	4.0 (1)	16.7 (3)	
Sector of current practice				
Governmental/Semi-governmental	86.0 (37)	96.0 (24)	72.2 (13)	**0.026**
Private	14.0 (6)	4.0 (1)	27.8 (5)	

* Calculated using chi-square test and independent *t*-test. *p*-value < 0.05 was considered statistically significant. Bold indicates that *p*-value reached statistical significance.

**Table 2:** Survey questions related to ADHD screening.

	% (n)
Have you completed any specific training for diagnosis and/or treatment of ADHD?	
Yes	53.5 (23)
No	46.5 (20)
Do you use a tool to screen for ADHD symptoms?	
Yes	86.0 (37)
No	14.0 (6)
Do you use the Conners Rating Scale to screen for ADHD symptoms?	
Yes	54.1 (20)
No	45.9 (17)
Do you use the Vanderbilt Assessment Scale to screen for ADHD symptoms?	
Yes	67.6 (25)
No	32.4 (12)
Do you use the Child Behavior Checklist to screen for ADHD symptoms?	
Yes	16.2 (6)
No	83.8 (31)
Do you use the Behavior Assessment System for Children to screen for ADHD symptoms?	
Yes	10.8 (4)
No	89.2 (33)
What questions do you ask upon ADHD screening?	
Major symptom domains (inattention, impulsivity, hyperactivity)	88.4 (38)
Administer rating scales or questionnaires containing the DSM-5	51.2 (22)
Whether these symptoms cause impairment	81.4 (35)
MDT assessment	2.3 (1)
Full medical, family, social, academic history	7.0 (3)
Difficulties & challenges in their life./Academic reports	4.7 (2)
Who is involved in the clinical interviews for evaluation?	
Patient only	9.3 (4)
Patient and Parents/Custodian/Caregiver	76.7 (33)
Teacher involved	14.0 (6)
During the clinical interview, do you usually ask about all 18 symptoms listed in the DSM-5 with the patients or their parents?	
Yes	55.8 (24)
No	44.2 (19)
Do you discuss ADHD symptoms, such as the number of symptoms, duration, age at onset, setting (e.g., home, school), with the patient or parent during the clinical interview?	
All	65.1 (28)
Missing at least one	34.9 (15)
On average, how many cases per week do you usually screen for ADHD?	
1–5 cases	60.5 (26)
6–10 cases	30.2 (13)
More than 11 cases	9.3 (4)

**Table 3:** Survey questions related to ADHD treatment.

	% (n)
Frequency of the major challenges experienced when treating individuals with ADHD	
Lack or absence of early screening ADHD programs implemented	65.1 (28)
Physician-related factors	27.9 (12)
Family-related factors	46.5 (20)
Lack of available medication on the physician’s desk	51.2 (22)
Extended referral waiting list	44.2 (19)
Other factors (e.g., Lacks community resources and professionals trained specifically to deal with ADHD cases, adults refusing to consent for further history from patents/family members, lack of qualified treatment, some parents refusing to give the medicine when it is described to their children or giving the children less than they supposed to have)	16.3 (7)
How often do you request follow-up appointments for individuals with ADHD?	
Always	55.8 (24)
Often	16.3 (7)
Very often	23.3 (10)
Rarely	4.7 (2)
How often do you refer individuals with ADHD to rehabilitation services (e.g., Physical Therapy, Occupational Therapy)?	
Always	11.6 (5)
Often	18.6 (8)
Very often	16.3 (7)
Rarely	39.5 (17)
Never	14.0 (6)
If you refer patients to rehabilitation services, what is the main reason for your referral?	
Manage ADHD symptoms only	51.2 (22)
Other (e.g., Sensory processing, physical injuries (e.g., burn, fracture, trauma), musculoskeletal-related pain)	27.9 (12)
Missing	20.9 (9)
What class of medication do you prescribe for patients with ADHD?	
Stimulant (Methylphenidate/Amphetamines)	81.4 (35)
Non-stimulant (Atomoxine/Clonidine/Bupropion/Risperidone)	9.3 (4)
Not eligible to prescribe medications	9.3 (4)
Do you use Amphetamines to treat patients with ADHD?	
Yes	7.0 (3)
No	83.7 (36)
Missing	9.3 (4)
What are the treatment options that you offer to patients with ADHD?	
Pharmacological treatment only	20.9 (9)
Non-pharmacological treatment only (Cognitive behavioral therapy)	4.7 (2)
Combined treatment with (exercise, family training for skills to deal with their child, books and internet resources for self-help, diet, sleep, omega-3 supplement, Mindfulness Behavior therapy, education, speech therapy)	74.4 (32)

## Data Availability

Data are available upon reasonable request.

## Abbreviations

AAP	American Academy of Pediatrics
ADHD	Attention-deficit/hyperactivity disorder
AMPH	Amphetamine-based stimulants
CPGs	clinical practice guidelines
DSM-5	Diagnostic and Statistical Manual of Mental Disorders, Fifth Edition
HCP	healthcare professional
IRB	Institutional Review Board
MOH	Ministry of Health
MPH	Methylphenidate-based stimulants
NICE	National Institute for Health and Care Excellence
SFDA	Saudi Food and Drug Authority
SPSS	Statistical Package for the Social Sciences
